# Spatial and temporal variation in malaria transmission in a low endemicity area in northern Tanzania

**DOI:** 10.1186/1475-2875-5-98

**Published:** 2006-11-03

**Authors:** MJAM Oesterholt, JT Bousema, OK Mwerinde, C Harris, P Lushino, A Masokoto, H Mwerinde, FW Mosha, CJ Drakeley

**Affiliations:** 1 Joint Malaria Programme, Moshi, Tanzania; 2 Department of Medical Microbiology, Radboud University Nijmegen Medical Centre, Nijmegen, The Netherlands; 3 Kilimanjaro Christian Medical Centre, Moshi, Tanzania; 4 TPC Hospital, Moshi, Tanzania; 5 London School of Hygiene and Tropical Medicine, London, UK

## Abstract

**Background:**

Spatial and longitudinal monitoring of transmission intensity will allow better targeting of malaria interventions. In this study, data on meteorological, demographic, entomological and parasitological data over the course of a year was collected to describe malaria epidemiology in a single village of low transmission intensity.

**Methods:**

Entomological monitoring of malaria vectors was performed by weekly light trap catches in 10 houses. Each house in the village of Msitu wa Tembo, Lower Moshi, was mapped and censused. Malaria cases identified through passive case detection at the local health centre were mapped by residence using GIS software and the incidence of cases by season and distance to the main breeding site was calculated.

**Results:**

The principle vector was *Anopheles arabiensis *and peak mosquito numbers followed peaks in recent rainfall. The entomological inoculation rate estimated was 3.4 (95% CI 0.7–9.9) infectious bites per person per year. The majority of malaria cases (85/130) occurred during the rainy season (χ^2 ^= 62,3, p < 0.001). Living further away from the river (OR 0.96, CI 0.92–0.998, p = 0.04 every 50 m) and use of anti-insect window screens (OR 0.65, CI 0.44–0.94, p = 0.023) were independent protective factors for the risk of malaria infection. Children aged 1–5 years and 5–15 years were at greater risk of clinical episodes (OR 2.36, CI 1.41–3.97, p = 0.001 and OR 3.68, CI 2.42–5.61, p < 0.001 respectively).

**Conclusion:**

These data show that local malaria transmission is restricted to the rainy season and strongly associated with proximity to the river. Transmission reducing interventions should, therefore, be timed before the rain-associated increase in mosquito numbers and target households located near the river.

## Background

The incidence and clinical presentation of *Plasmodium falciparum *malaria shows considerable variation between age groups [1], countries [2,3] and relatively short distances in single areas [4]. A key parameter defining the variation is the malaria transmission intensity. Transmission intensity is commonly quantified by the entomological inoculation rate (EIR), the estimated level of exposure to malaria infected mosquitoes. Spatial and temporal variation in transmission intensity is often not included in EIR estimates. This variation is particularly important in areas of low transmission intensity where few infected mosquitoes are caught and focal *hotspots *of malaria transmission may exist. Low transmission areas often have an unstable EIR [1,4] with seasonal peaks that are commonly correlated with recent rainfall. Climatic [5-7], spatial [8-11], socio-economic [12-16] and other micro-environmental factors [13,14] are important contributors to heterogeneity of malaria transmission in small geographic areas. Within a village there can be clustering of mosquitoes in a certain regions [17] with large proportions of vectors collected in relatively few of the houses [18,19]. Similar to clustering of mosquitoes, clinical malaria episodes can also be clustered in individuals [12] or housing groups [12,16,20].

Factors that play a role in transmission can be studied using computerized mapping with global positioning systems (GPS) and geographical information systems (GIS) [10]. When environmental factors are coupled with clinical data, it may be possible to identify populations, households or areas that carry the heaviest burden of malaria or are the most important potential contributors to malaria transmission. Consequently, the impact of malaria control efforts can be maximized by implementing tailored control measures to carefully selected areas [20].

The study objective was to map malaria transmission patterns in a village in Lower Moshi, northern Tanzania, that is representative for this low transmission area. By determining micro-environmental factors that influence the risk of malaria, the aim was to identify high risk areas where transmission reducing interventions can be focused.

## Methods

### Study site

The study was conducted in the village of Msitu wa Tembo in lower Moshi (latitude 3° 33' S; longitude 37° 17' E) at an average altitude of 714 m. Lower Moshi lies between the Masai Savannah and foothills of Mount Kilimanjaro and is hypoendemic for falciparum malaria. Irrigation from the nearby Karanga river makes the area suitable for small scale farming which is the predominant occupation.

The average annual rainfall is 615 mm (10 year average) and is highly seasonal with the period March–May accounting for 70% of the annual precipitation. The remainder falls during the unpredictable short rains in October–December. Between these two rainy seasons are a hot dry season during January–February and a cool dry season during June–September.

This study was part of a larger study on malaria immunity and received ethical approval from the ethical committees of the National Institute for Medical Research, the Kilimanjaro Christian Medical Centre, and the London School of Hygiene and Tropical Medicine.

### Demography

All houses, roads and possible breeding sites were located with a hand-held GPS unit (Trimble GeoExplorer III). The program ArcView GIS 3.2 was then used to develop a map of the village and environs. Each house in the village was mapped, censused and its structure was evaluated. Rainfall data was collected from a nearby sugar plantation and measured daily at 21 points.

### Entomology

#### Mosquito light traps

Mosquitoes were collected in 10 houses that were selected to be representative of the different housing structures in the village. Mosquitoes were caught with a standard Centre for Disease Control light traps (CDC, Atlanta, GA, USA) every week for one year (January–December 2004). Traps were hung at the end of an occupied bed with a bed net that was newly provided by the investigators. Traps were set for 12 hours, from 7 pm to 7 am [22]. In the morning, traps were then collected and mosquito species determined and counted. Male *Anopheles *mosquitoes, *Culicines *and non-vector *Anophelines *were discarded.

#### CSP ELISA

Female *Anopheles *mosquitoes stored on silica gel for circumsporozoite protein (CSP) ELISA as described by Wirtz *et al*. [23]. Briefly, the head and thorax were removed for every mosquito and stored in an uncoated 96-well microtitre plate until the assays were performed. Samples were prepared individually and assayed in batches of four with positive batches re-assayed as single mosquitoes. Insectary reared unfed female *Anopheles *were used as negative controls with the kit supplied CSP antigen as positive control. Samples were read by eye and on an ELISA plate reader at 495 nm. The EIR and confidence intervals were calculated as described by Drakeley *et al*. [24]. A conversion factor was used to adjust for the difference between light trap catches and man biting catches [25], giving the formula: infectious bites per person per year = 1.605 * (number of positive mosquitoes/number of traps) * 365.

#### Mosquito speciation

The protocol described by Scott *et al*. [26] was followed. Individual mosquitoes were randomly selected from ELISA test plates and the DNA extracted using the salt-Tris-EDTA (STE) method. 50 μl of mosquito triturate was added to 100 μl of STE and mixed. Insectary reared *Anopheles gambiae ss*. were used as controls and PCR products were visualised using 1.5% agarose gels.

### Morbidity data

A government clinic in the village centre, that was the nearest health facility for ~8 km, was used for collecting morbidity data. Each person visiting the clinic with suspected malaria was sent to the laboratory for confirmation by microscopy. Giemsa-stained slides were read by trained microscopists. Information about age, sex and residence were collected from the persons with a positive blood film and morbidity data were linked to individual houses. Individuals were considered patients if they had more than five *P. falciparum *parasites per 200 white blood cells in combination with symptoms suggestive of malaria. If the time between two malaria episodes of an individual exceeded one month, both were considered as separate episodes. Individuals were treated with sulphadoxine-pyrimethamine as first line treatment, with amodiaquine as second line and quinine reserved for severe cases as directed by the local clinical officer and in accordance with national guidelines.

### Data analysis

Based on the rainfall and temperature data (not shown) the year was divided into three arbitrary seasons: dry (January–March), wet (April–June) and cool (July–December). The distance of individual households to the river was calculated in steps of 50 metres using ArcView GIS 3.2 software and subsequently categorised as close (<1,200 m), intermediate (1,200–1,600 m) and far (>1,600 m). Morbidity data and mosquito catches were summarized per week. Statistical analyses were performed in SPSS version 11.0.1 (SPSS Inc., Chicago, USA). Continuous variables were analysed using t-test, one-way ANOVA for normal distributed data or Wilcoxon rank sum test and Spearman's correlation coefficient for not normally distributed data. Categorical variables were analysed using chi-square tests. Parasite densities were analysed after log-transformation (LN). Logistic regression models were used to determine the association between malaria incidence and housing conditions, age and distance to the river. Age was included in these models as a categorical variable (<5 years, 5–14 years and ≥ 15 years old) and distance to the river as a continuous variable in steps of 50 meters.

## Results

### Demography

The village of Msitu consisted of 863 houses and a population of 3,388 individuals. Based on questionnaires used in an ancillary study (Shekelaghe et al *in press*), the vast majority of the population reside in the village permanently with travel outside largely restricted to the adult male population. The median age of the population was 17.0 years (IQR 7.0–30.0) and 51.1% (1,721/3,370) was male.

### Entomology

**Figure 1:**
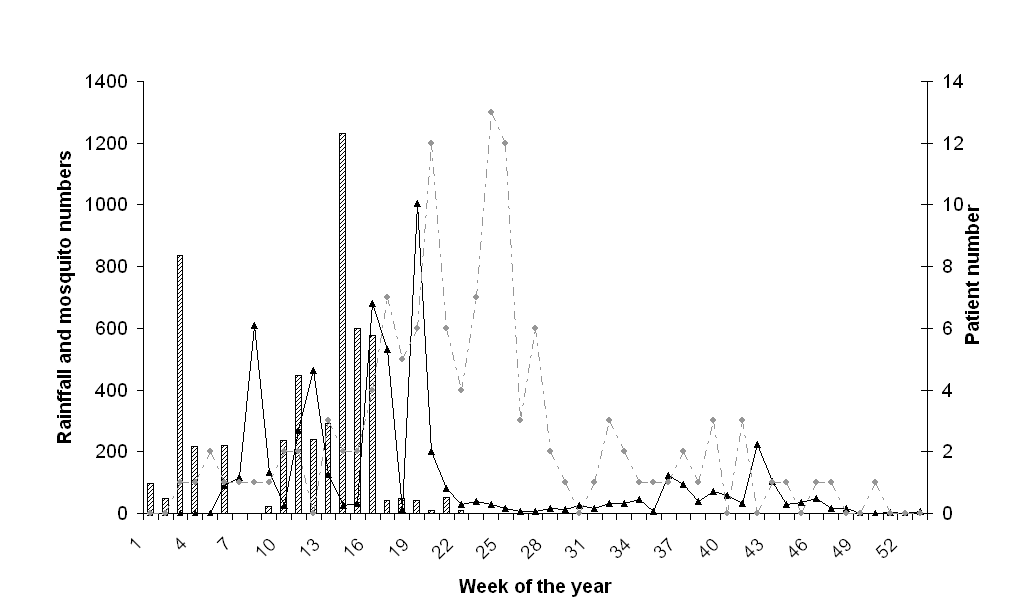
**Rainfall, numbers of mosquito and patients during 2004**. Bars indicate the total rainfall per week collected in 21 rain gauges at the nearby sugar plantation (left hand y-axis). The solid line indicates the total proportion of female *Anopheles *mosquitoes caught per week (left hand y-axis). The broken line the total number of malaria cases seen by the health centre per week (right hand Y-axis).

A total of 510 CDC light traps were set in Msitu and 12859 mosquitoes were caught. Of the mosquitoes caught, 44.5% were of the Culex (5,722/12,859) and 55,5% of the *Anopheles *genus (7,137/12,859), 79% of the latter were female (5,638/7,137). The seasonal relationship between rainfall, mosquito densities and malaria cases is shown in figure 1. There is a sequential progression of peaks in rainfall, number of female *Anopheles *mosquitoes caught and number of patients. No short rains (October – December) were experienced in the year that the study was conducted.

In the CSP ELISA three female *Anopheles *mosquitoes were positive: one in March and two in May. Two of these positive mosquitoes were caught in one house. Based on CSP ELISA data and the number of traps set (n = 510), an overall EIR of 3.43 (95% CI 0.7–9.9) infectious bites per person per year was calculated.

PCR amplification of mosquito samples was successful in 77.2% (61/79) of the randomly selected mosquitoes and indicated that all mosquitoes were of the *An. arabiensis *species (61/61).

The number of houses selected was insufficient to determine a possible relation with distance to the river. However, despite the limited number of houses, medium or large window size (Wilcoxon Rank-sum, p = 0.01) and the absence of window screens (Wilcoxon Rank-sum, p < 0.001) were associated with a higher number of caught female *Anopheles *mosquitoes in univariate analysis.

### Malaria morbidity

In the period January–December 2004, 130 malaria episodes were observed in 122 individuals. These individuals came from 105 households with three household experiencing more than two separate malaria episodes (three separate episodes in one man from a household of three individuals; four episodes in three individuals from a household of six individuals; and four episodes in different individuals from a household of twelve individuals). In the dry season 17 patients reported at the health facility, compared to 85 in the wet season and 28 in the cool season (χ^2 ^= 62,3, p < 0.001). In the dry season the geometric mean parasite density was 9309 (IQR 2,879–43,002), in the wet season 4575 (IQR 1,040–28,119) and in the cool season 2864 (IQR 579–30,264) parasites/μl.

### Factors associated to malaria incidence

**Figure 2:**
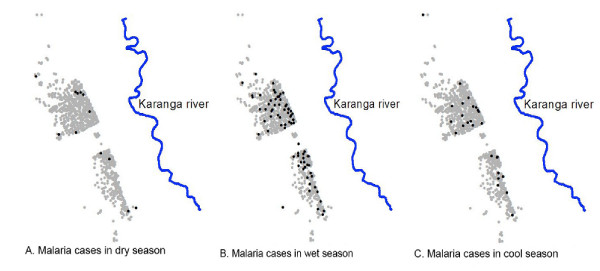
**Spatial distribution of malaria cases per season**. Grey dots represent the houses. Black dots represent the malaria cases. Blue line indicates the river.

The number of malaria cases was highest in houses close to the river that was considered as the main breeding site in the study area (Figure 2). The number of malaria cases was negatively associated to the distance to the river. Of those individuals who were living close to the river (<1,200 meters) 5.1% (71/1,379) presented at the health centre with parasites during the study period compared to 3.0% (45/1,500) of those living at 1,200–1,600 meter and 2.8% (14/509) of those living at >1600 meter from the river (χ^2 ^test for trend = 9.08, p = 0.0026). The proportion of cases living at <1,200, 1,200–1,600 and >1,600 meter from the river was not different for the different seasons (χ^2 ^= 0,94, p = 0.92). In addition to the distance to the river, three other factors appeared associated with malaria incidence (Table 1). There was a relation with age with malaria episodes most frequently occurring in children aged 5–14 years, followed by those below 5 years. The use of window screens resulted in a lower risk of malaria and malaria cases were more frequently observed in houses with larger windows, although the latter was not statistically significant. Other housing conditions such as roof type (p = 0.85), wall type (p = 0.90) and the presence of eaves (p = 0.32) were not related to risk of malaria.

**Table 1:** Risk factors associated with malaria incidence

	Malaria incidence			
	Crude OR	p-value	Adjusted OR	p-value
Distance to breeding site	0.95 (0.91–0.98)	0.003	0.96 (0.92–0.998)	0.04
				
Age				
under 5 yrs	2.44 (1.46–4.06)	0.001	2.36 (1.41–3.97)	0.001
5–14 yrs	3.80 (2.51–5.75)	< 0.001	3.68 (2.42–5.61)	<0.001
15 yrs and older	1.0 reference		1.0 reference	
				
Window screen				
Yes	0.586 (0.41–0.84)	0.03	0.65 (0.44–0.94)	0.023
No	1.0 reference		1.0 reference	
				
Window size				
Big/medium (<50 cm^2^)	1.431 (0.944–2.171)	0.092	1.328 (0.859–2.054)	0.202
Small/no window (>50 cm^2^)	1.0 reference		1.0 reference	

Crude and adjusted Odds Ratio (OR) are represented with a 95% confidence interval between brackets.

## Discussion

This study describes the pattern of malaria transmission in an area of very low transmission intensity, quantified by an estimated EIR of 3,43 (95% CI 0.7–9.9) infectious bites per person per year. The findings indicate a seasonal pattern of malaria transmission with a clear geographical clustering of malaria cases associated with proximity to the river.

The strong relation between malaria incidence and the distance to a river (as a potential breeding site) has been observed before [10,11]; that this relation is apparent within a single village where a relatively small number of malaria episodes were observed is noteworthy. Msitu wa Tembo is a semi-arid area, which provides few breeding sites associated with irrigated areas on the banks of the river. In non-irrigated semi-arid areas transmission is often below detection level during the dry season, but high toward the end of the rainy season [27]. This study shows a similar pattern with a marked effect of the rainy season and high mosquito numbers concentrated in the 2–4 weeks after the heavy rainfall. This increase is presumably the results of an increase in mosquito breeding sites where mosquitoes mature in 10–14 days after oviposition. It was expected that the association between distance to the river and malaria episodes would be strongest in the dry season when the river is the only available breeding site and would become weaker after the rains when alternative breeding sites are more abundant. There was however no difference in the geographical distribution of malaria cases in the different seasons. This may in part be due to the low number of malaria cases in the dry season and that the GIS analysis was not adjusted for proximity of other houses. However, these data suggest that riverine breeding sites are the major source of vectors throughout the year. The principal effect of rainfall may be to increase the number and size of these sites maintaining the observed association of malaria cases and distance to the river. These data need interpreting in light of variations in rainfall patterns; in the year of study the annual rainfall was less the an 50% of the 10 year average (280 mm vs 615 mm) with no short rains.

The strong seasonal pattern of malaria transmission that we observed is typical of low transmission areas with seasonal rainfall [1,4]. In this study, malaria infections are observed in all age groups, as previously described [1] although malaria episodes are most common in children below fifteen years of age. This may reflect a lower level of immunity but may be partly attributable to a relative underestimation of adult malaria cases as well. Adults may be more likely to self medicate and, as a result, passive case detection may give incorrect estimates of age related disease patterns. Passive case detection may have resulted in an underestimation of symptomatic malaria episodes overall and definitely of parasite carriage, that can occur without symptoms even in low endemic areas [28]. However, whilst acknowledging that not all malaria infections will lead to a visit to the health clinic, there is no other health facility within a 10 km radius and clinic attendance was free during the study period, its is probable that all malaria related visits have been included and consider it unlikely that this has biased our results substantially.

There appeared to be clustering of malaria cases in certain houses. Although numbers were too small to allow firm conclusions, two out of three infected mosquitoes were caught in the same house and some households experienced several malaria episodes. This may indicate memorized site fidelity, as was previously noted in a nearby field site for *An. arabiensis*[29]

These findings provide a basis for transmission reducing interventions. Improving housing conditions such as window size and window screening can reduce the risk of malaria. In this respect, it is important to note that the major vector in this area, *An. arabiensis *[30] is known to show exophilic behaviour. This behaviour presents obstacles for interventions with treated bed-nets and indoor residual spraying and calls for greater focus on strategies such as larval control [31] or mass administration with gametocytocidal drugs to reduce the human infectious prior to the transmission season[32]. The data presented here suggest that with appropriate seasonal and spatial targeting these can be more effectively implemented.

## Conclusion

The strong relation of malaria cases with the river suggest that focal malaria control is possible, either by larval control in river beddings or by selectively targeting households close to the breeding sites with transmission reducing interventions.

## Authors' contributions

MJAMO conducted the fieldwork, analysis and wrote the manuscript, JTB was involved in data analysis and manuscript preparation. OKM, CH, PL, AM and HM were involved in data-collection. FWM was involved in the study logistics and the planning of the study. CJD and JTB designed the study and were involved in data analysis and manuscript preparation.
